# Low expression of N-myc downstream-regulated gene 2 in oesophageal squamous cell carcinoma correlates with a poor prognosis

**DOI:** 10.1186/1471-2407-13-305

**Published:** 2013-06-24

**Authors:** Wei Cao, Guozheng Yu, Qiang Lu, Juliang Zhang

**Affiliations:** 1 Department of Interventional Radiology, Tangdu Hospital, The Fourth Military Medical University, No.1 Xinshi Road, Xi’an 710038 , Shaanxi, China; 2 Department of Interventional Radiology, Shaanxi Cancer Hospital, No.309 Yanta West Road, Xi’an 710061, Shaanxi, China; 3 Department of Thoracic Surgery, Tangdu Hospital, The Fourth Military Medical University, No.1 Xinshi Road, Xi’an 710038, Shaanxi, China; 4 Department of Vascular and Endocrine Surgery, Xijing Hospital, The Fourth Military Medical University, Xi’an 710032, Shaanxi, China

**Keywords:** NDRG2, Oesophageal squamous cell carcinoma (ESCC), Prognosis

## Abstract

**Background:**

It is currently unclear whether a correlation exists between N-myc downstream-regulated gene 2 (NDRG2) expression and oesophageal squamous cell carcinoma (ESCC). The aim of this study was to examine the underlying clinical significance of NDRG2 expression in ESCC patients and to investigate the effects of NDRG2 up-regulation on ESCC cell growth *in vitro* and *in vivo.*

**Methods:**

Immunohistochemistry was used to determine the level of NDRG2 expressions in ESCC tissue, which was then compared to specific clinicopathological features in the patient and tissue specimens. Factors associated with patient survival were analysed. Moreover, the effects of up-regulating NDRG2 expression on the growth of an ESCC cell line were examined by MTT, colony formation, DNA replication activity and nude mouse model assays.

**Results:**

Notably low expression of NDRG2 in ESCC patients was inversely associated with clinical stage, NM classification, histological differentiation and patients’ vital status (all *P <* 0.05). ESCC patients expressing high levels of NDRG2 exhibited a substantially higher 5-year overall survival rate than NDRG2-negative patients. Furthermore, NDRG2 over-expression reduced the proliferation, colony formation and DNA replication activity in ESCC cells, as well as inhibiting the growth of ESCC cells *in vivo*.

**Conclusion:**

The present experiments demonstrated that NDRG2 may be a diagnostic and prognostic marker in patients with ESCC, and up-regulation of NDRG2 might act as a promising therapeutic strategy for aggressive ESCC.

## Background

Oesophageal carcinoma is regarded as the eighth most common malignant cancer and sixth most frequent cause of cancer death worldwide [1]. Oesophageal squamous cell carcinoma (ESCC) is the most common pathological type in developing nations, particularly in China [2-4]. It has been reported that there are 167,200 cases of oesophageal cancer in China each year, out of a global total of approximately 310,400 cases [5]. Despite improvements in its detection, surgical resection, and adjuvant therapy, the 5-year overall survival rate for oesophageal cancer is approximately 15-24% [6]. Current chemo/radiotherapy conditions act sub-lethally, but they cannot effectively suppress the proliferation of ESCC cells. Thus, a deeper understanding of the molecular mechanisms involved in the high rate of proliferation and significant invasion of ESCC cells will allow for the development of an adjuvant therapy to improve current treatment options.

NDRG2, a member of the N-myc downstream-regulated gene family, belongs to the alpha/beta hydrolase superfamily. It was first cloned at our university from a normal human brain cDNA library by subtractive hybridization (GenBank accession no. AF159092) and is regarded as a tumor suppressor gene that is transcriptionally repressed by c-Myc [7-9]. Accumulated evidence indicates that NDRG2 is down-regulated or undetectable in many human cancers [10,11]. Recently, it has been shown that breast cancer cells have low or undetectable NDRG2 expression, compared with high levels in normal tissues [11]. Further studies have found that NDRG2 is able to inhibit proliferation and enhance apoptosis in many malignant tumors [12]. In addition, NDRG2 could inhibit breast cancer angiogenesis by up-regulating p53 and down-regulating of VEGF [13]. These findings suggest that the expression of NDRG2 is inversely related to cell proliferation, especially in terms of cancer cell proliferation. However, the effects of NDRG2 expression in ESCC remain unclear.

The objective of this study was to investigate NDRG2 expression and its clinical significance in ESCC and to further explore the effects of NDRG2 up-regulation on ESCC cell growth. In this study, western blot analysis and immunohistochemistry methods were used to examine NDRG2 expression. The correlation of NDRG2 expression with clinicopathological features specific to ESCCs was also assessed. Furthermore, using an adenovirus NDRG2 expression system, we verified the effects of Ad-NDRG2 on proliferation, clone formation number, DNA replication activity of Eca-109 cells, and the growth of tumors in a nude mouse model.

## Methods

### Patient information and tissue specimens

This study was approved by the Ethics Committee of the Fourth Military Medical University, and all patients agreed to participate in this study. Fresh oesophageal squamous cell carcinoma specimens were collected from 143 patients at the Xijing Hospital and Tangdu Hospital of the Fourth Military Medical University (Xi’an, China) from 2003 to 2005. ESCC tissues were obtained from resected tumors and confirmed by pathological review. ESCC specimens were staged in accordance with the American Joint Cancer Committee/Union for International Cancer Control (AJCC/UICC) classification guidelines. The grading and histopathology subtyping of ESCC specimens were based on WHO criteria.

### Immunohistochemistry

Immunohistochemical staining was performed to assess NDRG2, cyclinD1 and Ki67 protein expression, as described previously [14]. For immunohistochemistry, formalin-fixed tumor tissues were embedded in paraffin, and serial 4 μm sections were obtained using a Leica microtome. For staining, tumor sections were deparaffinised in toluene, rehydrated in an alcohol gradient, and permeabilised in citrate buffer (pH 6.0). Sections were then quenched with 3% H_2_O_2_ for 5 min to eliminate endogenous peroxidase activity and washed in PBS. Sections were incubated overnight with different antibodies, followed by NDRG2, cyclinD1 or Ki67 antibodies incubation with a biotinylated goat anti-rat or anti-rabbit IgG antibody for 15 min. After washing, sections were incubated with streptavidin peroxidase, lightly counterstained with hematoxylin, and observed under a photomicroscope.

### Staining evaluation

Fresh oesophageal squamous cell carcinoma specimens were collected from 143 patients at the Xijing Hospital and Tangdu Hospital of the Fourth Military Medical University (Xi’an, China) from 2003 to 2005. NDRG2 expression was detected in all specimens. Tissue specimens were examined separately by 2 pathologists under double-blinded conditions. The molecular expression was scored as positive if >10% of cells had moderate-to-strong staining. Expression was scored as negative if either cytoplasmic or membranous staining were noted in < 10% of cells or if neither cytoplasmic nor membranous staining were observed [15].

### Cell lines and reagents

Normal human oesophageal epithelial cell line HEEC and five ESCC cell lines (EC8712, KYSE30, Eca-109, KYSE70 and KYSE150) were obtained from American Type Culture Collection(ATCC, USA) and maintained in RPMI 1640 medium (Invitrogen, USA) supplemented with 10% foetal bovine serum (FBS, Life Technologies, USA), 100 units/ml penicillin G sodium (Sigma, USA), and 100 μg/ml streptomycin sulphate (Sigma, USA). Cells were grown at 37°C in a humidified atmosphere containing 5% CO_2_. A mouse anti-human NDRG2 monoclonal antibody (1:1000 dilution) was purchased from Abcam (UK). A rabbit anti-human β-actin monoclonal antibody (1:3000 dilution) was purchased from Biomics Corporation (China). MTT and western blot kits were from Sigma.

### Immunofluorescence assay

Cells were fixed in 4% paraformaldehyde for 30 min at room temperature and permeabilised with PBS containing 0.1% Triton X-100 for 10 min. After washing 3 times with PBS, cells were incubated with 50 μl of NDRG2 primary antibody (1:200) at 4°C overnight. Then, the cells were incubated with CY3 (1:400) at room temperature for 2 h before applying the mounting medium (containing DAPI (Sigma; 1:100) for nuclear counterstaining). Cells were washed three times with PBS before observation. The results were analysed using a fluorescence microscope (Olympus).

### Gene infection

A multiplicity of infection (MOI) of 40 was determined experimentally for Eca-109 cells. Cells were seeded in 6-well plates at a density of 5 × 10^5^ cells/well and grown to approximately 80% confluence. After removing the medium, adenovirus-expressing NDRG2 (Ad-NDRG2) or the negative control gene LacZ (Ad-LacZ) was added in serum-free 1640 medium, incubated for 2 h, replaced with fresh 1640 supplemented with 10% FBS and incubated for 48 h.

### Western blot

For whole-cell extracts, cells were washed with ice-cold PBS and collected by scraping. Cell pellets were homogenised in extraction buffer (50 mM Tris–HCl, 0.1% SDS, 150 mM NaCl, 100 mg/ml phenylmethylsulfonyl fluoride, 1 mg/ml aprotinin, 1% NP-40, and 0.5% sodium orthovanadate), incubated at 4°C for 20 min and then centrifuged for 20 min at 12,000 rpm. Protein levels in the extracts were quantified using the Bio-Rad DC protein assay. For western blots, 80 μg of whole-cell extract was resolved on 12% SDS-polyacrylamide gels, then transferred onto nitrocellulose membranes (0.45 μm, Millipore, USA) in 25 mM Tris-base, 190 mM glycine, and 20% methanol using a semi-dry blotter. Membranes were blocked with 5% fat-free milk and 0.1% Tween-20 in Tris-buffered saline (TBS). Primary antibodies were used at the concentration recommended by the suppliers. Detection of monoclonal and polyclonal antibodies was performed using horseradish peroxidase-conjugated goat anti-mouse/anti-rabbit immunoglobulins, respectively, and an enhanced chemiluminescence (ECL) substrate.

### Cell proliferation assay

Cell growth following infection was evaluated by an MTT assay. Cells were seeded in a 96-well plate (1 × 10^3^ cells/well) and incubated for different time periods. At different time points post-infection, the cells were incubated with 0.5 mg/ml MTT (Sigma). Four hours post-infection, the medium was replaced with 150 μl dimethyl sulfoxide (DMSO) (Sigma) and vortexed for 10 min. Absorbance (A) was then recorded at 570 nm using an Easy Reader 340 AT plate reader (SLT-Labinstruments, Salzburg, Austria). Relative optical density (OD) values were calculated as percentages of the control. All experiments were performed three times independently.

### Plate colony formation assay

For colony formation assays, 1 × 10^3^ cells infected with different adenovirus constructs were seeded into 60 mm dishes with 5 ml of 1640 medium supplemented with 10% FBS. After 10 days, the resulting colonies were rinsed with PBS, fixed with methanol at -4°C for 5 min, and stained with Giemsa (Sigma) for 20 minutes. Counting was performed only on clearly visible colonies (diameter > 50 μm).

### DNA replication activity assay

DNA replication activity was examined using BrdU (5-Bromo-2-deoxyUridine). Cells grown on coverslips (Fisher) were infected with Ad-LacZ and Ad-NDRG2. Two days after infection, the cells were incubated with BrdU for 1 h and stained with an anti-BrdU antibody (Roche) according to the manufacturer’s instructions. The cells were then cultured in the mounting medium (containing DAPI (Sigma, 1:100) for nuclear counterstaining). Cells were washed three times with PBS before observation. The results were analysed using a fluorescence microscope (Olympus).

### Xenograft study in nude mice

For inoculation into nude mice, Eca-109 cells were washed with PBS, digested with trypsin, and resuszpended in serum-free 1640 medium. After centrifugation (800 rpm), cell pellets were suspended in 1640 medium. The cell suspension (5 × 10^6^ cells in a volume of 100 μl PBS) was injected subcutaneously into the hind legs of 4-week-old female BALB/C athymic (nu/nu) mice (SLAC Laboratory Animal Company, Shanghai, China) [16]. When tumors reached a volume of 200 mm^3^, the mice were arbitrarily assigned to different groups (n = 6 each) to receive intratumoural injections of 10^9^ PFU Ad-NDRG2, Ad-LacZ, or PBS. Intratumoural injections were repeated every 3 days for a total of 24 days. Tumors were measured (perpendicular diameters) every day and their volumes calculated. On day 24, the mice were sacrificed, and their tumors removed for analysis. Tumor volumes were calculated based on calliper measurements of the length and width of the lesions using the following formula: 0.5 × length × width^2^. The growth curve was then derived from these data.

All experimental procedures were conducted in accordance with the Detailed Rules for the Administration of Animal Experiments for Medical Research Purposes issued by the Ministry of Health of China and received ethical approval by the Animal Experiment Administration Committee of the Fourth Military Medical University (Xi’an, P. R. China). All efforts were made to minimise the animals’ suffering and to reduce the number of animals used.

### Statistical analysis

Experiments *in vitro* were performed 3 times, and each experiment was performed in triplicate. Data from all quantitative assays are expressed as the mean ± standard deviation (SD) and were analysed statistically using a one-way ANOVA and the independent-samples *t-*test. In the *in vivo* study, associations between NDRG2 expression and categorical variables were analysed by using the chi-square test or the Fisher exact test, as appropriate. Correlations between NDRG2 expression and categorical variables were analysed by using the Spearman correlation test. Survival curves for both NDRG2-high and NDRG2-low expression patients were plotted using the Kaplan–Meier method, and statistical differences were compared using a log-rank test. Differences with *P* < 0.05 were considered statistically significant.

## Results

### Association between decreased expression of NDRG2 and progression of ESCC

To further examine whether expression of the NDRG2 protein is linked to the clinical progression of ESCC, the following samples were subjected to IHC staining with a human NDRG2 antibody: 143 paraffin-embedded, archived ESCC tissue samples, including 5 cases of stage I, 64 cases of stage IIA, 23 cases of stage IIB, 38 cases of stage III and 13 cases of stage IV tumors. The mean age of the 143 ESCC patients was 62 years (range 38 to 86 years), and follow-up data were available for all patients.

The results of IHC staining are summarised in Table 1. The NDRG2 protein was highly expressed in 60 of 143 (42.0%) human ESCC samples. Statistical analyses showed no relationship between patient gender or age and NDRG2 expression (Table 2). However, NDRG2 expression decreased progressively through tumor stages I to IV. Moreover, NDRG2 expression in ESCC tissues with poor differentiation was statistically significantly lower than that in well or moderately differentiated ESCC tissues. The data revealed that NDRG2 expression was strongly associated with clinical stage (*P* = 0.009), T classification (*P* < 0.0001), N classification (*P* < 0.0001), M classification (*P* = 0.036) and histological differentiation (*P* = 0.005) (Table 2). Spearman analysis also revealed a correlation between NDRG2 expression and the clinical stage (r = −0.299, *P* < 0.0001), T classification (r = −0.347, *P* < 0.0001), N classification (r = −0.387, *P* < 0.0001), M classification (r = −0.170, *P* = 0.042) and histological differentiation (r = −0.268, *P* = 0.001) (Table 3). Taken together, these observations support the hypothesis that the progression of ESCC is associated with decreased NDRG2 expression.

**Table 1:** Clinicopathological characteristics of patient samples and expression of NDRG2 in ESCC 143

**Variable**	**Number of cases (%)**	**Variable**	**Number of cases (%)**
Gender		Cigarette smoking	
Male	95 (66.4)	No	51 (35.7)
Female	48 (33.6)	Yes	92 (64.3)
Age(years)		Alcohol drinking	
≤60	78 (54.5)	No	37 (25.9)
>60	65 (45.5)	Yes	106 (74.1)
Clinical stage		T classification	
I	5 (3.5)	T1	9 (6.3)
IIA	64 (44.8)	T2	52 (36.4)
IIB	23 (16.1)	T3	63 (44.1)
III	38 (26.6)	T4	19 (13.2)
IV	13 (9.0)	Histological differentiation	
N classification		Well	45 (31.4)
N0	61 (42.7)	Moderate	50 (35.0)
N1	69 (48.3)	Poor	48 (33.6)
N2	13 (9.0)	Expression of NDRG2	
M classification		Negative	83 (58.0)
M0	130 (90.9)	Positive	60 (42.0)
M1	13 (9.1)	Location	
Therapy		Upper	17 (11.9)
Surgery only	80 (55.9)	Middle	80 (55.9)
Surge + CT/RT/CRT	63 (44.1)	Lower	46 (32.2)
Complication			
Yes	35 (24.5)		
No	108 (75.5)		

**Table 2:** Correlation of NDRG2 expression with clinical histopathological characteristics in 143 ESCC specimens

**Variable**	**NDRG2 expression**		***p***
	**Negative (%)**	**Positive (%)**	
Gender			
Male	57 (60.0)	38 (40.0)	0.154^a^
Female	26 (54.2)	22 (45.8)	
Age(years)			
≤60	50 (64.1)	28 (35.9)	0.127 ^a^
>60	33 (50.8)	32 (49.2)	
Cigarette smoking			
No	33 (64.7)	18 (35.3)	0.289 ^a^
Yes	50 (54.3)	42 (45.7)	
Alcohol drinking			
No	17 (45.9)	20 (54.1)	0.121 ^a^
Yes	66 (62.3)	40 (37.7)	
Clinical stage			
I	1 (20.0)	4 (80.0)	0.009^b^
IIA	29 (45.3)	35 (54.7)	
IIB	15 (65.2)	8 (34.8)	
III	28 (73.7)	10 (26.3)	
IV	10 (76.9)	3 (23.1)	
T classification			
T1	1 (11.1)	8 (88.9)	<0.0001^b^
T2	23 (44.2)	29 (55.8)	
T3	43 (68.3)	20 (31.7)	
T4	16 (84.2)	3 (15.8)	
N classification			
N0	22 (36.1)	39 (63.9)	<0.0001^b^
N1	50 (72.5)	19 (27.5)	
N2	11 (84.6)	2 (15.4)	
M classification			
M0	72 (55.4)	58 (44.6)	0.036^a^
M1	11 (84.6)	2 (15.4)	
Histological differentiation			
Well	18 (40.0)	27 (60.0)	0.005^b^
Moderate	30 (60.0)	20 (40.0)	
Poor	35 (72.9)	13 (27.1)	

^a^ Statistical analyses were done by the Fisher’s exact test.

^b^ Statistical analyses were done by the Pearson’s chi square test.

A *P* value of < 0.05 was considered significant.

**Table 3:** Spearman correlation analysis between NDRG2 expression and clinical pathologic factors

**Variable**	**NDRG2 expression**	***p ***^**a**^
	**Correlation coefficient (*r***_***s***_***)***	
Gender	-.121	.124
Age(years)	-.135	.109
Cigarette smoking	.101	.232
Alcohol drinking	-.145	.084
Clinical stage	-.299	.0001
T classification	-.347	.0001
N classification	-.387	.0001
M classification	-.170	.042
Histological differentiation	-.268	.001

^a^ The Spearman correlation test was used for statistical analyses. *P* values < 0.05 were considered statistically significant.

### Association between expression of NDRG2 and overall survival of ESCC patients

**Figure 1:**
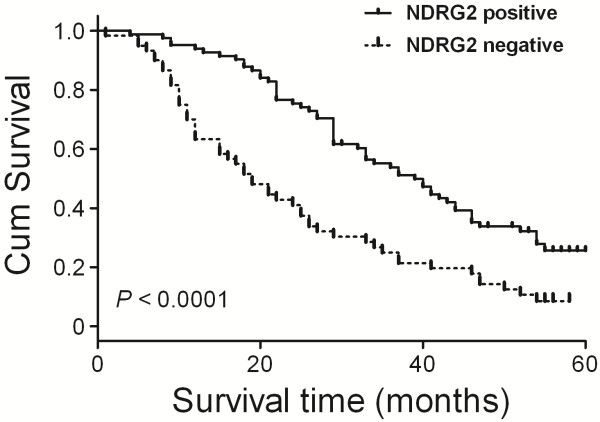
**Correlation of the overall survival rate of ESCC patients with NDRG2 expression pattern.** Curves were estimated using the Kaplan-Meier method (*P* < 0.0001). Continuous line represents NDRG2 positive group; dotted line represents NDRG2 negative group.

A log-rank test and Kaplan-Meier analysis were used to calculate the effect of NDRG2 on survival. The log-rank test showed that NDRG2 protein expression was strongly related to patients’ survival time (*P* < 0.0001; Figure 1). More specifically, the median survival time of patients with high NDRG2 protein expression levels was 39months, whereas the median survival time of those with low NDRG2 levels was only 19 months. The cumulative 5-year survival rate was 36.1% in the high NDRG2 expression group, whereas in the low NDRG2 expression group, the survival rate was only 6.7%.

### Up-regulation of NDRG2 expression by Ad-NDRG2 in Eca-109 cells

**Figure 2:**
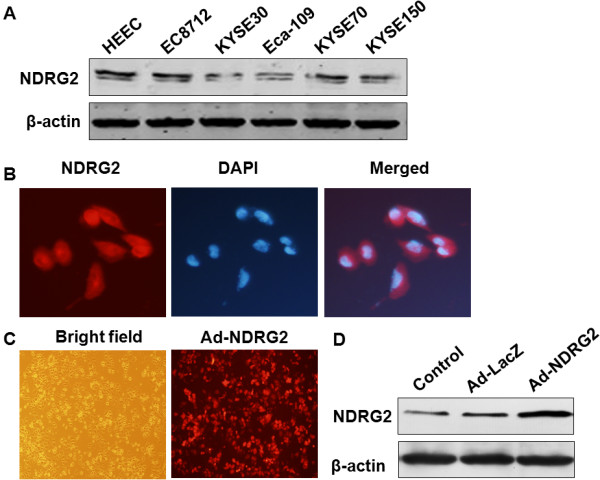
**Up-regulation of NDRG2 expression by Ad-NDRG2 in Eca-109 cells. A**. HEEC cells (a normal human oesophageal epithelial cell line) and five ESCC cell lines (EC8712, KYSE30, Eca-109, KYSE70 and KYSE150) were collected for protein extraction and analysed for NDRG2 expression using western blot. **B**. Subcellular localisation of NDRG2 in Eca-109 cells. Eca-109 cells were fixed and incubated with a primary anti-NDRG2 monoclonal antibody and a CY3 secondary antibody. Red fluorescence (NDRG2) was observed in the cytomembrane and cytoplasm of Eca-109 cells. Blue fluorescence indicates DAPI nuclear staining. **C**. Immunofluorescence showed the efficiency of Ad-NDRG2 infection (red). **D**. Western blot analysis of NDRG2 in Eca-109 cells infected with Ad-NDRG2. Equal amounts of protein (80 μg/lane) were subjected to western blot analysis as described in the Methods.

To examine NDRG2 expression in ESCC cell lines, we detected NDRG2 protein expression in the normal human oesophageal epithelial cell line HEEC and five ESCC cell lines (EC8712, KYSE30, Eca-109, KYSE70 and KYSE150) by western blot. The results showed that the expression of NDRG2 was the highest in the HEEC cell line, followed by the EC8712, KYSE70 and KYSE150 cell lines, and it was the lowest in the Eca-109 and KYSE30 cell lines (Figure 2A). To further determine the role of NDRG2, we chose to use Eca-109 (low endogenous NDRG2 levels) as our experimental model in the following studies. An adenovirus-NDRG2 construct was designed to increase the expression levels of NDRG2. Moreover, it was shown that subcellular localisation of NDRG2 was observed in the cytoplasm of Eca-109 cells (Figure 2B). The infection efficiency of Ad-NDRG2 in Eca-109 cells was also examined (Figure 2C). Next, western blot was used to evaluate NDRG2 protein expression up-regulation. Compared to the Ad-LacZ group (the negative control), the expression of NDRG2 was successfully increased in Eca-109 cells after infection with Ad-NDRG2 (Figure 2D).

### Inhibitive effects of up-regulating NDRG2 on the growth of Eca-109 cells

**Figure 3:**
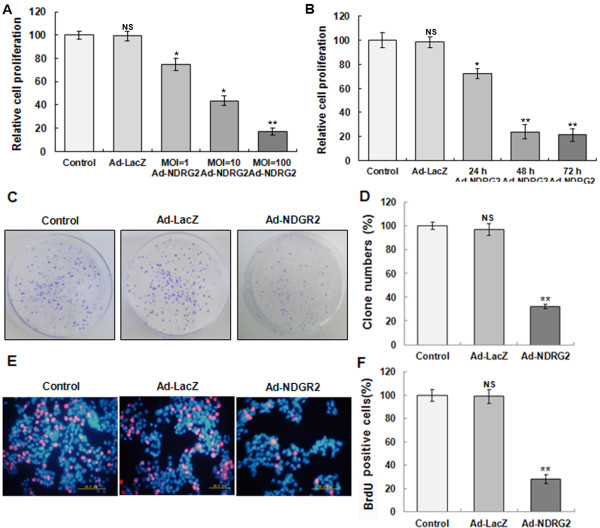
**Up-regulating NDRG2 inhibits Eca-109 cell growth. A**. Dose-dependent suppression of Eca-109 cell proliferation by Ad-NDRG2. Eca-109 cells were infected with various amounts of Ad-NDRG2 or Ad-LacZ (at MOI of 1, 10, and 100) and incubated for 48 h. Cell proliferation was detected by MTT assay. **B**. Time-dependent suppression of Eca-109 cell proliferation by Ad-NDRG2. After Ad-NDRG2 or Ad-LacZ infection at an MOI of 100, the cells were incubated for different time periods (24, 48 and 72 h). Cell proliferation was detected by MTT assay. **C** and **D**. The effect of Ad-NDRG2 on Eca-109 cell colony formation was examined. After Ad-NDRG2 or Ad-LacZ infection, the cells were incubated for approximately 10 days until visible cell colony formation. Only the clearly visible colonies (diameter > 50 μm) were counted. **E** and **F**. The effect of Ad-NDRG2 on Eca-109 cell DNA replication activity was examined. After Ad-NDRG2 or Ad-LacZ infection, the cells were incubated for 2 days and cultured with BrdU for 1 h and DAPI for nuclear counterstaining. The results were analysed using a fluorescence microscope; BrdU positive (red) cells were counted. Data are mean ± SD from three independent experiments. Statistical significance was assessed using a one-way ANOVA and Student’s *t*-test. * or ** indicates *P* < 0.05 or *P* < 0.01, respectively, compared to the control.

To investigate the inhibition of Eca-109 cell growth by up-regulating NDRG2, MTT, colony formation and DNA replication activity assays were examined. First, an MTT assay was designed with an MOI gradient (1, 10, and 100) and infection time gradient (day 1, day 2, and day 3). We infected cells with Ad-NDRG2 or Ad-LacZ at an MOI of 1, 10, and 100; after 48 h treatment, the proliferation of the Ad-LacZ group was not significantly different compared to the control. On the contrary, the inhibition ratio of the Ad-NDRG2 group was augmented by increasing the Ad-NDRG2 concentration (Figure 3A). There was also a clear time dependence of Ad-NDRG2 efficacy, with only moderate inhibition of proliferation at 24 h and maximal inhibition achieved at 48 h. Extending the incubation period to 72 h showed no further increase in the inhibitory efficacy (Figure 3B). Next, we assayed the contribution of Ad-NDRG2 in Eca-109 cell colony formation. The different groups were incubated for 2 weeks and counted. As shown in Figure 3C and 3D, compared with the control, the colony formation ratio of Ad-NDRG2-treated cells decreased significantly to only 33.6%. In contrast, there was no obvious difference in the colony formation ratio between the control and Ad-LacZ groups. Last, the cell DNA replication activity was examined using BrdU, a synthetic thymidine analogue that binds to replicating DNA. We performed a BrdU assay and found that, after 3 h incubation with BrdU, the cells in the DNA replication phase presented a red colour. As shown in Figure 3E and 3F, compared to the control, the red cell ratio of Ad-NDRG2-treated cells was just 28.6%. There was no obvious difference in the DNA replication activity between the control and Ad-LacZ groups. These data revealed that NDRG2 overexpression could inhibit the growth of Eca-109 cells.

### Suppression of tumor growth in a nude mouse model by intratumoural Ad-NDRG2 injection

**Figure 4:**
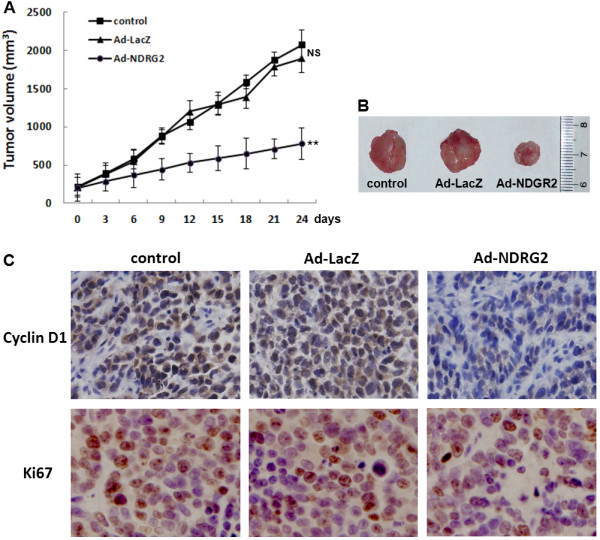
**Effects of intratumoural Ad-NDRG2 injections on the growth of Eca-109 cells xenografted into mice.** Eca-109 cells (5 × 10^6^) were injected into the upper hind limb of athymic nude mice and allowed to grow until the tumour volume reached 200 mm^3^. Mice then received intratumoural Ad-NDRG2 or Ad-LacZ injections (at 50 MOI) or PBS (20 μl as control) every 3 days. **A**. Tumour growth curve. The tumour growth was assessed every 3 days until Day 24 of treatment by measuring two perpendicular diameters and calculating the volume in mm^3^. Statistical analysis was performed on Day 24 values only, using a one-way ANOVA and Student’s *t*-test. ** indicates *P* < 0.01 as compared to the control. **B**. Representative photographs of xenografted tumours are shown. **C**. Intratumoural the protein expression was assessed by cyclinD1-immunolabeling (400 × magnification) or Ki67-immunolabeling (400 × magnification) on paraffin-embedded Eca-109 cell tumour sections. Representative images are shown. Statistical analysis was carried out using a one-way ANOVA. The results are shown as the mean ± SD of three different fields per mouse from a total of six mice. **P < 0.01.

To investigate the effects of Ad-NDRG2 on tumor growth *in vivo*, we injected 1 × 10^9^ PFU Ad-NDRG2, Ad-LacZ, or PBS every 3 days into pre-established human Eca-109 ESCC tumours (approximately 200 mm^3^) grown in nude mice. As shown in Figure 4A and B, the Ad-NDRG2 group achieved a sustained and significant arrest of tumour growth (66.7% decrease in mean tumour volume on day 24 compared with the control group), whereas the growth of tumours injected with Ad-LacZ was not significantly inhibited (3.8% decrease compared with the control group). We sacrificed the mice 24 days after beginning intratumoural injections and removed the tumours for the protein expression analysis (Figure 4C). Immunolabelling with cyclinD1 and Ki67 were examined in tumours excised from the mice. Moreover, the cyclinD1 and Ki67 expression levels in the Ad-NDRG2 group were decreased dramatically compared with the control group. There was no significant difference between the control and Ad-LacZ groups.

## Discussion

Although surgical resection and adjuvant therapy are commonly used in oesophageal carcinoma patients, the overall survival rate of oesophageal cancer remains very low. The development of biological therapies for oesophageal carcinoma is therefore urgently required. Earlier studies have shown that NDRG2, a new tumour suppressor gene, is lowly expressed and correlates with the progression of breast cancer [17]. However, the correlation of NDRG2 with ESCC and the effect of NDRG2 on ESCC cell growth are currently unclear. In this study, we analysed the correlation of NDRG2 expression with clinical stage, TNM clinical classification, histological differentiation and survival time in the ESCC patients. Moreover, we used an adenovirus-mediated gene expression technology to up-regulate NDRG2 in the ESCC cell line, Eca-109, to directly investigate the effect of NDRG2 on cell growth *in vitro* and *in vivo*.

Members of the human NDRG family, comprising NDRG1, NDRG2, NDRG3 and NDRG4, share 57%-65% amino acid identity [18]. Although we do not yet fully understand the roles of the NDRG family members, it is possible that these proteins have a crucial function in tumour progression and differentiation. Previous studies have shown that NDRG2 was highly expressed in adult brain and skeletal muscle and almost undetectable in some human cancer lines [19-21], suggesting that it may play important functions in different tissues. It was reported that the expression level of NDRG2 mRNA was very high in brain, salivary gland, skeletal muscle and mammary gland; low in bone marrow, testis, peripheral blood and placenta; and not detectable in leukocytes, colon and some tumour cell lines [22-24]. Recent reports have shown that NDRG2 was differentially expressed in tumour and normal tissues, and NDRG2 expression was consistently down-regulated in grade Ш meningioma at both the transcriptional and translational levels [25]. Further studies have found that NDRG2 inhibits the proliferation, adhesion and invasion of breast cancer cells [26,27]. In addition, a previous study reported that NDRG2 could inhibit the metastatic potential of breast cancer cells, specifically via the suppression of CD24 or MMP-9 expression. Tumour angiogenesis in breast cancer was inhibited by overexpression of NDRG2 up-regulating the expression of p53 and VHL and down-regulating the expression of VEGF and HIF-1α [28]. NDRG2 could suppress cell proliferation possibly through the following mechanisms: first, NDRG2 could regulate cyclin D1 and T-cell factor (TCF)/β-catenin activity, both of which are critical signaling pathways in cell growth [29,30]; second, NDRG2 could suppress nuclear factor kappa B (NF-kB) activity, suggesting a possible mechanism for NDRG2 to participate in carcinogenesis and progression of human malignancy [31,32]; third, silencing NDRG2 attenuates p53-mediated apoptosis, and p53 could increase NDRG2 expression to promote tumor cell apoptosis, suggesting that NDRG2 also suppresses tumor cell proliferation through the p53-mediated apoptosis signaling pathway [12].

The expression and effects of NDRG2 in ESCC are unclear. In this paper, our data suggest that in ESCC tissues, NDRG2 expression decreased progressively through tumour stages I to IV, and NDRG2 expression in the well-differentiated ESCC tissues was significantly high. The data revealed that NDRG2 expression was strongly associated with the TNM clinical stage. Importantly, an overall survival analysis revealed that positive NDRG2 expression was correlated with longer survival time in ESCC patients. Lastly, *in vitro* and *in vivo* NDRG2 up-regulation could inhibit Eca-109 cell proliferation, decrease the clone formation number and the DNA replication activity of Eca-109 cells. Moreover, in a nude mouse model of ESCC, Ad-NDRG2 treatment achieved a sustained and significant arrest of tumour growth, and expression of cyclinD1 and Ki67 was lower in tumours excised from mice in the Ad-NDRG2 group.

## Conclusion

In summary, our results provide evidence that NDRG2 contributes to the growth of ESCC. NDRG2 expression correlates with ESCC TNM stage, tumour differentiation and overall survival of ESCC patients, and up-regulating NDRG2 expression by adenovirus could inhibit the growth of ESCC cells *in vitro* and *in vivo*. Thus, our study provides evidence that NDRG2 may play an important role in the development, differentiation, and carcinogenesis of ESCC and could therefore be utilised in diagnosis and as a prognosis indicator in ESCC patients.

## Competing interests

The authors declare that they have no competing interests.

## Authors’ contributions

WC carried out the experimental design, data analysis and interpretation, manuscript writing; GY carried out the immunoassays; QL carried out the cellular study; JZ carried out the study concept and design, obtained funding, critical revision of the manuscript for important intellectual content and study supervision. All the authors read and approved the final manuscript.

## Pre-publication history

The pre-publication history for this paper can be accessed here:

http://www.biomedcentral.com/1471-2407/13/305/prepub
